# A First Diastolic Function Evaluation in the Personalized Exercise Prescription Program for Solid Organs Transplanted Subjects: Is Atrial Strain Useful?

**DOI:** 10.3390/jpm15010032

**Published:** 2025-01-17

**Authors:** Melissa Orlandi, Marco Corsi, Vittorio Bini, Roberto Palazzo, Stefano Gitto, Claudia Fiorillo, Matteo Becatti, Marco Maglione, Laura Stefani

**Affiliations:** 1 Sport Medicine Centre, Clinical and Experimental Medicine, University of Florence, 50134 Florence, Italy; melissa.orlandi@unifi.it (M.O.); marco.corsi@unifi.it (M.C.); roberto.palazzo@unifi.it (R.P.); 2 Medicine Department, University of Perugia, 06100 Perugia, Italy; binivittorio@gmail.com; 3 Internal Medicine and Hepatology, Department of Experimental and Clinical Medicine, University of Florence, 50134 Florence, Italy; stefano.gitto@unifi.it; 4 Department of Experimental and Clinical Biomedical Sciences “Mario Serio”, University of Florence, 50134 Florence, Italy; claudia.fiorillo@unifi.it (C.F.); matteo.becatti@unifi.it (M.B.); 5 CV Ultrasound Division, ESAOTE Spa, 50127 Florence, Italy; marco.maglione@esaote.com

**Keywords:** atrial strain, solid organ transplantation, diastolic function

## Abstract

**Background/Objectives**: Solid organ transplant recipients (OTR) have been recently involved in exercise prescription programs in order to reduce the high prevalence of cardiovascular diseases. The normal systolic and diastolic cardiac function is fundamental to personalizing the prescription. Diastolic dysfunction can be associated to a higher risk of cardiovascular events and left atrial (LA) strain is an emerging parameter in the evaluation of diastolic compromising, especially in subjects with preserved ejection fraction. Left atrial (LA) strain has never been explored in this category. The study aimed to evaluate the contribution of the LA strain in the assessment of diastolic function of OTR and its potential contribution in the exercise program. **Methods**: 54 solid OTR (liver and kidney transplants) regularly trained for at least 12 months in a home-based, partially supervised model at moderate intensity estimated by cardiopulmonary exercise test, underwent a complete echocardiographic analysis. The measured variables included left ventricle systolic function (ejection fraction, EF), diastolic function (E/A and E/E’), LA indexed volumes, LA peak atrial longitudinal strain (PALS) and LA peak atrial contraction strain (PACS). The data were compared to those of 44 healthy subjects (HS). **Results**: The OTR showed an overweight condition (BMI: 25.79 ± 2.92 vs. 22.25 ± 2.95; *p* < 0.01). Both groups showed a preserved systolic function (EF: OTR 63.1 ± 3.5% vs. HS 66.9 ± 6.1; *p* < 0.001), while diastolic standard parameters were significantly different (E/A, 1.01 ± 0.4 vs. 1.96 ± 0.74; *p* < 0.001; E/E’, 9.2 ± 2.7 vs. 6.9 ± 1.3; *p* < 0.001, in OTR and HS respectively) despite being normal. LA strain was significantly lower in OTR vs. HS (4C PALS, 33.7 ± 9.7 vs. 45.4 ± 14.19; *p* < 0.001; 4C PACS, 15.9 ± 6.7 vs. 11.6 ± 7.5; *p* = 0.006; 2C PALS, 35.3 ± 11.1 vs. 47.6 ± 14.9; *p* < 0.001; 2C PALS, 17.4 ± 4.9 vs. 13.2 ± 14.97; *p* = 0.001; in OTR and HS respectively). A specific correlation of two- and four-chamber PACs and PALs with BMI has been observed (R for 4C PALS −0.406 ** and 2C PALS −0.276 *). **Conclusions**: These findings suggest that the coexistence of increased bodyweight in asymptomatic OTR patients can exacerbate the impairment of LA strains. LA strain detection could be useful in the development of a personalized exercise program for OTRs, especially for asymptomatic subjects and those with elevated cardiovascular risk profile, to potentially manage the exercise program in the long term. Larger studies will confirm the role via an eventual structured clinical score index.

## 1. Introduction

Exercise prescription represents a non-pharmacological treatment for several non-communicable diseases [1,2,3,4] due to its peculiar mechanisms of reducing the inflammation process and the cardiovascular risk. Solid organ-transplanted subjects are an emerging category that has recently involved in the personalized exercise programs [5,6,7] that are conducted in parallel with drug treatments. After transplantation, mortality risk for acute events is higher if compared to the general population [8]. Many causes can be recognized, and among them the prolonged period of sedentarism with the long exposure to multiple pharmacological treatments are the most important aspects. Tailored exercise prescription therefore requires special attention to the cardiovascular systo-diastolic parameters, and also to the body composition, especially in those subjects suffering from a subtle multiorgan dysfunction. Diastolic dysfunction is in fact associated with increased cardiovascular mortality [9], and the modifications of atrial deformation properties are related to the atrial function being compromised [10]. Atrial strain is one of the emerging parameters used in the evaluation of diastolic function, particularly in the categorization of subjects with preserved ejection fraction (EF) [11,12]. A correct cardiological screening is therefore important for them [13], along with the importance of evaluating the diastolic function, especially in obese or overweight patients [14]. Multiple echocardiographic parameters are used to differentiate and to identify the diastolic dysfunction [15], however, no data regarding atrial strain analysis have been investigated, especially in this category. A new proposal for the modification of the current diastolic guidelines is assessed. Diastolic function evaluation is going to be implemented, and strain analysis represents one of the most recently parameters considered [16]. In addition, considering that diastolic dysfunction can be associated with a preserved or reduced systolic function, as reported for many chronic diseases [17,18,19], but no data or experiences related to transplants have been reported. The study is aimed at evaluating the diastolic function closely via specific parameters, ultimately assessing the atrial function in asymptomatic liver and kidney transplant recipients regularly following an exercise prescription program. The data were compared to those of a healthy active group. This is considered in the context of optimizing the tailored exercise prescription program in this category.

## 2. Materials and Methods

### 2.1. Protocol Study

A group of 54 solid organ transplant recipients (OTR), 39 with liver and 15 with kidney transplants (14 women and 40 men, mean age 59.43 ± 8.75 years old), were retrospectively investigated and enrolled in the study from 2021 up to 2023. They were clinically stable, asymptomatic, and showed no recent arrhythmic acute events. The causes of liver transplantation were previous HCV liver cirrhosis (85%), or in the other cases, sclerosing cholangitis (7%) or primary biliary cirrhosis (8%). In the group with kidney transplantation, the main causes were a history of polycystosis (60%), pyelonephritis (2%), glomerulonephritis (8%), Berger’s nephropathy (5%) and congenital single kidney (15%). All subjects were screened for a tailored mixed (aerobic and counter-resistance) exercise program established at moderate power. The exercise prescription consisted of mixed physical activity (aerobics and counter-resistance) at least 3 times a week, with 30 min of aerobics at an intensity of about 60% of the maximal heart rate. The aerobics session was followed by counter-resistance exercises including at least 8 groups of body muscles. It was suggested to train via fast walking in the range of the heart rate identified, or alternatively to follow specific training classes. The modality of training could also include online registered classes, available by mobile phone using a specific QR code. In this case, the exercise program prescribed could be considered a “home-based program”, which was partially supervised. All OTR were included after at least 12 months of the personalized exercise prescription program. The intensity, duration and frequency of exercise were estimated by cardiopulmonary exercise test (CPET) in all subjects, and planned following the general ACSM guidelines [17]. Exercise was conducted at at least the first aerobic threshold, established by the VO2 value [20].

The control group was composed of 44 healthy active subjects (HS) who regularly trained and did not follow a specific exercise prescription program. In any case, the level of physical activity in both groups was assessed and compared using the IPAQ questionnaire [21], which also helped in confirming the adherence to the program for OTR. The Karvonen Formula was used to calculate the aerobic exercise, and adjusted for the eventual use of beta-blockers [22].

### 2.2. Inclusion/Exclusion Criteria

Comorbidities, such as diabetes, arterial hypertension or other metabolic diseases, were not a reason for exclusion. The included transplanted subjects had mild or moderate hypertension and were on antihypertensive treatment (calcium channel-blockers, alpha-blockers, ACE inhibitors or ARBs) and immunosuppressive therapy, including drugs such as calcineurin inhibitors (Ciclosporin or Tacrolimus), in combination with Mycophenolate or Everolimus and steroids (Methylprednisolone).

The exclusion criteria were having acute cardiovascular symptoms or recent acute cardiovascular events, or having absolute contraindications that prevented the practicing of physical activity.

The local Independent Ethics Committee approved the study (study ID: ISRCTN66295470, Tuscany, Italy), which was developed according to the ethical parameters established in the Declaration of Helsinki (1964) and its later amendments [23].

### 2.3. Echocardiographic Exam

All the subjects were submitted to an echocardiographic exam. The myocardial function was assessed by transthoracic echocardiography using the MyLABX8 Esaote echocardiograph (Esaote, Florence, 50127, Florence, Italy) equipped with a cardiac probe single Cristal Px15 for adults at 1–5 MHz.

Two certified cardiologists were involved in the acquisition of the images, and the average value after at least 3 repeated measurements were used. The standard systolic and diastolic parameters were considered following the ASE guidelines [24]. For the standard parameters, the following measures were considered: diameters (left ventricle end-diastolic diameter (LVEDD) and thicknesses (interventricular septum (IVS) and posterior wall (PW)) of the left ventricle, LV cardiac mass, indexed left atrial volume (iLAV)). Regarding the diastolic function, the mitral inflow pattern (wave E/A ratio, deceleration time), annular mitral septal and lateral TDI with E/E’ ratio were considered. The mitral E/E’ ratio was calculated and considered as a reliable index of LA and filling pressures. Diastolic function was classified as normal or abnormal with impaired relaxation (grade 1), pseudo-normal (grade 2), restrictive (grade 3) or indeterminate range [15]. Particularly for the last class of diastolic function described, the additional values of tricuspid regurgitation were measured for a correct interpretation. In the case of valvular heart disease, its severity was evaluated according to the ASE 2015 guidelines [24].

For the quantification of LA size, 2C and 4C LA size views were captured excluding the pulmonary veins and LA appendage from the LA tracing. The plane of the mitral annulus was used as the inferior border. A mild enlargement of the LA was defined as LA volume ≥ 29 mL/m^2^, a moderate one as ≥34 mL/m^2^, and a severe one as ≥40 mL/m^2^ [25]. The images for the strain calculation were captured and later processed by use of the X-strain Speckle Tracking software (ESAOTE X-Strain LA F13) included in the echo-MyLab and dedicated to the specific chamber strain quantification. Among the inclusion criteria, a quite good acoustic window, suitable for the acquisition of echocardiographic parameters, was fundamental. The LA dimensions were measured at the end of the LV systole when this chamber had reached its maximum size during cardiac cycle, as recommended [25].

All subjects completed the echocardiographic examination with the evaluation of myocardial deformation parameters, using a dedicated software (XStrain TM-ESAOTE-Genoa, Italy) specific to the atrial strain.

According to the criteria established in the EACVI/ASE consensus document [26], it was possible to obtain the overall study and the average of myocardial deformation by acquiring the images in full cycle and at a high frame rate. Particularly for the atrial strain analysis, starting from the QRS marker, after cycle acquisition, the two phases of reservoir and contraction, peak atrial longitudinal strain (PALS) and peak atrial contraction strain (PACS), measures of atrial reservoir and atrial active conduit, respectively, were considered, as reported in the literature [27,28].

### 2.4. Cardiopulmonary Test

The CPET was conducted on the basis of the guidelines [17,18,19,20,29,30,31] using an electromagnetic brake cycle ergometer (Ergoline, ERGOLINE ergoselect 100 P, Livorno, 57121 Livorno (Italy)) and a specific gas measurement machine (COSMED Quark CPET, Albano Laziale, 00141, Rome, Italy). Each participant was invited to avoid strenuous physical training the day before the test, and to abstain from consuming solid foods or carbohydrate-rich drinks for three hours before the test. The test was performed in the morning under controlled conditions (temperature, 18–24 °C; humidity, 30–60%). The ramp protocol for cardiopulmonary testing was tailored based on gender and body composition to aim for muscle exhaustion at between 8 and 12 min. An oro-facial mask connected to a gas-measuring device was used. Exhaled CO_2_ and O_2_ consumption were measured breath by breath. The lowest possible increase in watts (1, 2, or 5 watts) was set for each ramp to achieve the most linear increase in load and, therefore, a more physiological response. After 3 min of warming up by cycling without load at 50 rpm, the test followed the following steps: at the start of the actual effort, cycling was required at a cadence between 60 and 80 rpm until muscle exhaustion. The test concluded when the participant could no longer maintain their cycling cadence despite verbal encouragement. The test was considered maximal if at least two of the following criteria were met: Respiratory Exchange Ratio (RER) > 1.10, maximum heart rate > 85% according to age, and a plateau oxygen consumption (increase <150 mL·min^−1^) in the last 30 s of the test. The test was stopped early in the presence of cardiovascular signs and symptoms (complex ventricular arrhythmias, drops in systolic blood pressure, dizziness, etc.). Continuous monitoring included a 12-lead ECG and oxygen saturation. During the test, various parameters were measured, including oxygen consumption (VO2), carbon dioxide production (VCO2), tidal volume (VT), respiratory rate (RF), minute ventilation (VE), heart rate (HR), and workload (WR). The lactate threshold was determined using the V-slope and ventilator equivalents approach. Other variables analyzed included the relationship between oxygen consumption and heart rate (VO2/HR, a measure of stroke volume), the relationship between oxygen consumption and workload (VO2/W slope, a measure of circulatory efficiency), and the product of VO2 peak (mL/kg/min) and systolic blood pressure (a measure of circulatory strength). For the present investigation, the VO2 max value was considered as an expression of a normal heart’s performance in the two groups.

### 2.5. Statistical Analysis

The Shapiro–Wilk test was used to assess the normal distribution of data. Due to their asymmetric distribution, differences between groups were tested by the Mann–Whitney’s U-test and relationships between variables by the Spearman correlation coefficient. All calculations used IBM-SPSS^®^ version 26.0 (IBM Corp., Armonk, NY, USA, 2019). A two-sided *p*-value < 0.05 was considered significant.

## 3. Results

Data are expressed as mean ± standard deviation and are expressed in Table 1. The BMI values were higher in the OTR group (OTR^BMI^ 25.79 ± 2.92 vs. HS^BMI^ 22.25 ± 2.45 with *p* < 0.001). Both groups showed a sufficiently active level of physical activity, although this was significantly higher in HS (IPAQ: OTR 1053.87 ± 1024.30 vs. HS 1974.45 ± 1438.87 METs/week, *p* = 0.02); OTR maintained the same adherence to the initial IPAQ level. Regarding the CPET parameters, only the VO2 max was considered to plan the exercise program, and therefore to estimate the differences. The value of VO2 max was significantly diverse in the two groups; however, both were in the normal range (VO2 max: 22.90 ± 7.30 vs. 38.87 ± 28.65, *p* = 0.01; VO2 max (%): 85.3 ± 23.62 vs. 91.74 ± 12.43, *p* = 0.02, in OTR and HS respectively).

Regarding the echocardiographic parameters, the main morphological (IVS, PW, LVEDD) and systolic (EF) parameters were normal, without significant differences between the groups. The preserved EF has also been confirmed in OTR.

Following diastolic evaluation guidelines [15], diastolic function was interpreted on the basis of an algorithm based on four parameters. All subjects had a normal systolic function, and tricuspid regurgitation was at low velocity, less than 2.8 msec. The other diastolic parameters considered were in the normal range for both groups, although the E/E’ ratio showed a higher value in OTR, despite not yet being pathological (E/E’ septum 9.93 ± 3.06 vs. 6.91 ± 1.31, *p* < 0.001; E/E’ lateral 7.63 ± 4.66 vs. 5.11 ± 1.34, *p* = 0.02, in OTR and HS respectively). The classification of the diastolic function was considered normal if at least three parameters were normal. The mean values of the E/E’ measured at the septum and lateral wall levels were 8.52 ± 3.32 for OTR and 5.92 ± 1.5 for HS. The E/E’ ratio was associated with a prolongation of relaxation time, as in the case of slight diastolic impairment. Considering the normal EF, all the variables included in the grading of the diastolic function are in the normal range. No eventual indetermined diastolic pattern could be recognized [15]. The absolute LA strain value, obtained by 4 and 2C, was in the range of normality for all and not significantly different between both, even if it was slightly higher in the healthy active subjects (HS) compared to the OTR.

**Figure 1:**
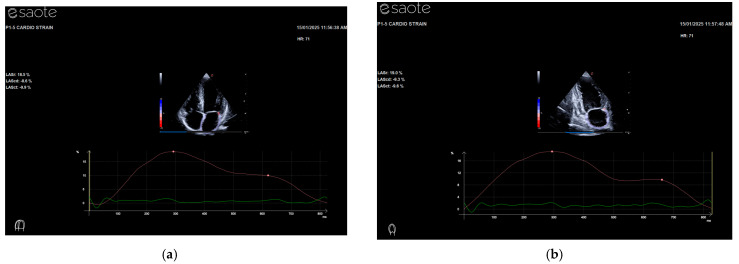
Demonstration of LA longitudinal myocardial deformation dynamics by 2-dimensional speckle-tracking echocardiography. A representative case of a subject from 4- (**a**) and 2- (**b**) chamber views. PALS indicates peak atrial longitudinal strain and PACS indicates peak atrial contraction strain (red line). Green line is the ECG trace that starts with QRS wave.

More evidence regarding the diastolic function has emerged from the correlations of the LA strain parameters (PALS and PACS from 4C and 2C, Figure 1) with all the other variables considered. A significant association of the LA strain has been shown with values of the standard echo diastolic function, despite these not yet being pathological (Table 2). This confirms that the major sensibility of the atrial longitudinal strain (PALS) in the early phase of diastolic impairment is not detectable by the standard evaluation approach, at least in this category (Table 2). Particularly evident is the correlation of PALS with BMI in asymptomatic OTR in the presence of normal systo-diastolic function. This aspect underlines major reservoir impairment in the presence of overweight.

The analysis of the correlation of the same parameters with all the variables in the two groups considered separately, shown as the correlation of the LA strain, is maintained in OTR (Table 3), particularly in the contraction phase (PACS).

## 4. Discussion

Personalized exercise is one of the most crucial points of the non-pharmacological treatment in many non-communicable chronic diseases, especially in those in whom the cardiovascular risk is high [1,2,3,4,5,6,7]. Transplanted subjects are a peculiar category with higher cardiovascular mortality if compared to the general population, despite the transplantation. Systolic function has also been largely studied by strain analysis [32], as given the importance of detecting potential cardiotoxicity due to the long-term exposure to the immunosuppressive therapy [5,6,7] and the necessity of maintaining a range of mixed exercise. On the contrary, the diastolic function, strongly related to mortality and fundamental to the physiological response for the adequation of cardiac adaptation, has not been extensively studied in this category. The elevated coexistence of comorbidities in transplanted patients induces us to focus on the potential impact of the parameters related to prolonged sedentariness, like BMI and body composition [33]. The most recent cardiovascular parameters linked with diastolic impairment, such as left atrial strain, could be relevant. Previous studies by MRI [34] have investigated this new aspect, referring to the importance of highlighting the atrial function, especially in patients with type 2 diabetes and overweight. The authors have underlined the importance of focusing on atrial deformation parameters by echocardiography [35] as indicators of augmented arrhythmic risk, as well as general cardiovascular risk [36]. Specific studies have also been performed on the use of the atrial strain to better identify and stratify the grade of diastolic dysfunction [37], especially in cases of different levels of heart failure. A closer correlation of global PALS compared to E/E’ ratio has been demonstrated in patients with moderate reductions in LV function. In this area, the present investigation is in agreement with the aims of previous studies. Some data are available in the literature, particularly regarding the category of male and female, highly trained athletes [38]; however, no specific results have been discussed in the context of the exercise prescription program, and therefore the frailty of subjects engaged in moderate-intensity exercise [39].

Despite the limits of the present investigation, due to the small sample investigated, the different ages of the subjects, and the incomplete evaluation of the strain, as a result of which some data of the conduit phase are missing, the results obtained are in agreement with the current literature; this explains the reduced LA reservoir and pump found in OTRs and the evident correlation with BMI. Understanding the clinical impact in terms of the daily use of the LA strain in an outpatient setting will necessitate a more accurate analysis, including a long-term evaluation. The potential inclusion of the LA strain in a dedicated multiparametric analysis for OTR should be considered to better investigate the effects of a reduction in the exercise tolerance period, particularly when the systolic function is preserved and the exercise program needs to be modified or implemented.

Few data are available on the effects of supervised exercise prescription programs on transplanted subjects, although these programs seem to be improved. The literature does not report any data regarding the potential improvement in diastolic function following exercise prescription with or without supervision. The OTR population often faces some difficulties in consistently adhering to exercise prescription programs; the major obstacle is not being completely conscious of the importance of non-pharmacological treatment, although this aspect is constantly underlined in medical counseling.

### Limitation of the Study

Some limits are present in the study. The major one regards the different ages of the two groups investigated. Despite this, the control group matches the age range of the main subjects (over 35 years old). This age range is, in terms of sport medicine, considered to be at the same level of cardiovascular risk as the general population, particularly as regards the prevalence of the coronary artery disease risk and initial diastolic dysfunction. The comparison, therefore, has been considered sufficiently adequate to evaluate this aspect. In addition, the two groups have been selected on the basis of the absence of cardiovascular events, and therefore these inclusion criteria should be set to reasonably reduce the potential negative effects of the different ages.

Another limit is represented by the absence of atrial conduit data. In fact, peak atrial longitudinal strain (PALS) and peak atrial contraction strain (PACS) are measures of the atrial reservoir and atrial active conduit, respectively. Despite this, the interval between the first phase (PALS) and the second phase (PACS), called diastasis, is an interval corresponding to the specific conduit phase. It is a plateau or diastasis because there is a balance between blood input and output, during which the atrium volumes do not change. The conduit function of the atria is responsible for controlling the amount of blood that comes out from the atrial chambers, without their active participation. In this case, as a consequence, the left atrial volumes are within the normal range, but this specific parameter has not been considered. We reasonably believe that, when following this first approach, the missing data do not substantially modify the sense of the paper. In addition, no data are available in the literature on the normal range of this phase in transplanted subjects not regularly trained.

Another limit is, at present, the impossibility of expanding this study to long-term follow-up with constant adhesion to the exercise programs proposed. This aspect is actually out of the range of the present investigation. Incomplete adhesion could be partially resolved by the suggestion of registered exercise lessons facilitated by mobile phone, or by following a short period of supervised exercise alternating with unsupervised, home-based exercise, as previously investigated [7,13]. Future studies will be in any case necessary to address these aspects.

## 5. Conclusions

The modulation and personalized management of the intensity of physical activity considered as a therapeutic effect, as a drug, in OTR is of recent interest in sports medicine. This is mostly relevant because of the lack of dedicated guidelines in this category, and the strong potential negative effects of sedentariness in the case of frailty. The systolic deformation parameters of the LV chamber are largely used for implementing myocardial function evaluations, especially in cases of suspected cardiotoxicity, particularly in the presence of metabolic chronic diseases. No data are available on the extensive use of the LA strain in patients with augmented cardiovascular profiles, such as OTR following an exercise prescription program. The data obtained are suggestive of a role of the LA strain in the cardiovascular risk stratification of overweight subjects, for whom long-term evaluation is essential.

## 6. Future Perspectives

The evaluation of diastolic disfunction and failure is important for subjects with frailty. Diastolic disfunction is associated with a measured risk profile, especially in cases of an unhealthy lifestyle with increased BMI. The importance of developing modes of atrial strain analyses is growing. Transplanted subjects have been largely studied exclusively for left ventricle systolic performance; at present, no data regarding the use of diastolic parameters in the long-term follow-up period have been proposed. The atrial strain is a new parameter of uncommon use in ambulatory settings, and it could be proposed in the future for use in the management of individualized exercise.

## Figures and Tables

**Table 1:** General and echocardiographic data.

	OTR (n = 54)	HS (n = 44)	*p*
Age (y)	59.43 ± 8.75	36.52 ± 12.24	<0.001
BMI (kg/m^2^)	25.79 ± 2.92	22.25 ± 2.45	<0.001
IPAQ (METs/week)	1053.87 ± 1024.30	1974.45 ± 1438.87	0.02
VO2 max (mL/kg/min)	22.90 ± 7.30	38.87 ± 28.65	0.01
VO2 max (%)	85.3 ± 23.62	91.74 ± 12.43	0.02
IVS (mm)	9.81 ± 1.1	9.49 ± 0.94	Ns
PW (mm)	9.61 ± 1.22	9.30 ± 0.86	Ns
LVEDD (mm)	50.52 ± 4.60	49.31 ± 3.61	Ns
EF (%)	64.39 ± 5.37	67.09 ± 6.44	0.05
LVMI (gr/m^2^)	100 ± 21.4	87.62 ± 14	Ns
E/A	1.01 ± 0.44	1.96 ± 0.74	<0.001
IVRT (ms)	85.67 ± 20.26	71.83 ± 26.60	0.01
DTc (ms)	219.15 ± 79.00	195.86 ± 38.47	Ns
E/E’ sep	9.93 ± 3.06	6.91 ± 1.31	<0.001
E/E’ lat	7.63 ± 4.66	5.11 ± 1.34	0.02
E/E’ med	8.52 ± 3.32	5.92 ± 1.5	0.03
iLAV (mL)	24.32 ± 8.65	27.22 ± 11.25	Ns
TR vel (m/s)	2.1 ± 0.4	1.9 ± 0.6	Ns
4C PALS (%)	33.45 ± 9.57	45.36 ± 14,19	<0.001
4C PACS (%)	15.88 ± 6.80	11.95 ± 7.48	0.003
2C PALS (%)	35.45 ± 11.26	47.55 ± 14.97	<0.001
2C PACS (%)	17.29 ± 5.09	13.16 ± 6.79	0.001

Legend: BMI, body mass index; VO2 max and VO2 max (%), absolute and relative oxygen volume consumption; IVS, interventricular septum; PW, posterior wall; LVEDD, left ventricle end-diastolic diameter; EF, ejection fraction; LVMI, left ventricle cardiac mass; IVRT, isovolumic relaxation time; DTc, deceleration time; E/E’ sep, septum E/E’; E/E’ lat, lateral E/E’; E/E’ med, median E/E’; iLAV, indexed left atrial volume; TR vel, tricuspid valve regurgitation; PALS, peak atrial longitudinal strain; PACS, peak atrial contraction strain in 2- and 4-chamber views. Ns: not significant.

**Table 2:** Correlations of the LA strain parameters with echocardiographic and anthropometric data.

	4C PALS	*p*	4C PACS	*p*	2C PALS	*p*	2C PACS	*p*
Age	−0.436 **	0.000	0.224 *	0.029	−0.496 **	0.000	0.338 **	0.003
BMI	−0.406 **	0.000	0.086	0.410	−0.276 *	0.013	0.188	0.101
IVS	−0.009	0.933	0.035	0.737	0.000	0.999	0.132	0.254
PW	0.113	0.266	0.228 *	0.026	0.097	0.394	0.187	0.103
LVDD	0.076	0.458	0.174	0.092	0.101	0.374	0.141	0.221
EF	0.184	0.069	0.025	0.808	0.080	0.478	0.118	0.305
E	0.040	0.696	−0.190	0.066	0.287 **	0.010	−0.198	0.085
A	−0.463 **	0.000	0.286 **	0.005	−0.362 **	0.001	0.295 **	0.009
E/A	0.378 **	0.000	−0.315 **	0.002	0.415 **	0.000	−0.346 **	0.002
IVRT	−0.291 **	0.004	0.062	0.552	−0.091	0.421	0.178	0.122
DTc	0.033	0.747	0.149	0.152	−0.016	0.885	0.171	0.136
E’ sep	0.267 **	0.008	−0.245 *	0.017	0.396 **	0.000	−0.376 **	0.001
E/E’ sep	−0.355 **	0.000	0.136	0.194	−0.336 **	0.002	0.227 *	0.048
E’ lat	0.206	0.169	−0.059	0.702	0.354 *	0.027	0.055	0.740
E/E’ lat	−0.574 **	0.000	−0.097	0.542	−0.253	0.143	0.130	0.456
iLAV	−0.028	0.786	−0.076	0.466	0.155	0.172	0.057	0.622
TR vel	−0.008	0.954	0.035	0.863	0.134	0.261	0.123	0.214
4C PALS	1.000		0.170	0.100	0.446 **	0.000	−0.094	0.416
4C PACS	0.170	0.100	10.000		−0.066	0.561	0.527 **	0.000
2C PALS	0.446 **	0.000	−0.066	0.561	10.000		0.183	0.111
2C PACS	−0.094	0.416	0.527 **	0.000	0.183	0.111	10.000	

Legend: BMI, body mass index; IVS, interventricular septum; PW, posterior wall; LVEDD, left ventricle end-diastolic diameter; EF, ejection fraction; IVRT, isovolumic relaxation time; DTc, deceleration time; E/E’ sep, septum E/E’; E/E’ lat, lateral E/E’; iLAV, indexed left atrial volume; TR vel, tricuspid valve regurgitation; PALS: peak atrial longitudinal strain; PACS, peak atrial contraction strain in 2- and 4-chamber views. *: Significance < 0.05. **: Significance < 0.001.

**Table 3:** Correlations and comparisons of the LA strain parameters with echocardiographic and anthropometric data of OTRs vs. HSs.

		OTR				HS			
		4C PALS	4C PACS	2C PALS	2C PACS	4C PALS	4C PACS	2C PALS	2C PACS
Age	R	0.000	−0.025	−0.233	−0.112	−0.347 *	0.108	−0.213	0.308
BMI	R	−0.243	−0.133	−0.159	−0.312 *	−0.202	0.092	−0.130	0.315
IVS	R	0.056	0.148	0.187	0.058	0.058	−0.161	−0.107	0.181
PW	R	0.048	0.227	0.236	0.143	0.311 *	0.154	−0.011	0.176
LVEDD	R	0.201	0.246	0.126	0.013	0.070	0.121	0.127	0.293
EF	R	0.221	−0.024	−0.122	0.148	−0.009	0.220	0.127	0.257
E/A	R	−0.057	−0.165	−0.002	−0.207	0.406 **	0.032	0.375 *	−0.006
IVRT	R	−0.090	0.004	0.015	−0.009	−0.247	−0.076	0.017	0.212
DTc	R	0.084	0.106	0.145	0.221	0.125	0.017	0.051	−0.067
E/E’ sep	R	−0.119	−0.148	−0.169	0.020	−0.081	0.027	0.190	0.138
E/E’ lat	R	−0.459	−0.262	−0.159	−0.090	−0.414 *	−0.105	0.060	0.038
iLAV	R	−0.263	−0.071	0.130	0.118	0.088	0.019	0.131	0.141
TR vel	R	−0.543	−0.075	0.154	0.126	0.021	0.035	0.125	0.165
4C PALS	R	1.000	0.488 **	0.215	0.376 *	1.000	0.183	0.338 *	−0.174
4C PACS	R	0.488 **	1.000	0.128	0.398 *	0.183	1.000	0.138	0.433 **
2C PALS	R	0.215	0.128	1.000	0.629 **	0.338 *	0.138	1.000	0.248
2C PACS	R	0.376 *	0.398 *	0.629 **	1.000	−0.174	0.433 **	0.248	1.000

Legend: BMI, body mass index; IVS, interventricular septum; PW, posterior wall; LVEDD, left ventricle end-diastolic diameter; EF, ejection fraction; IVRT, isovolumic relaxation time; DTc, deceleration time; E/E’ sep, septum E/E’; E/E’ lat, lateral E/E’; iLAV, indexed left atrial volume; TR vel, tricuspid regurgitation velocity; PALS, peak atrial longitudinal strain, PACS: peak atrial contraction strain in 2- and 4-chamber views. *: Significance < 0.05. **: Significance < 0.001.

## Data Availability

Data are available in the Sports Medicine Center’s dataset as part of sport and lifestyle reconditioning programs.
